# MRI-Based Radiomics to Predict Response to Neoadjuvant Therapy in Locally Advanced Rectal Cancer: A Retrospective Study

**DOI:** 10.3390/jpm16060282

**Published:** 2026-05-25

**Authors:** Ilaria Ambrosini, Roberto Francischello, Salvatore Claudio Fanni, Lorenzo Faggioni, Francesca Pia Caputo, Karolina Cwiklinska, Gayane Aghakhanyan, Emanuele Neri, Riccardo Lencioni, Dania Cioni

**Affiliations:** 1 Academic Radiology, Department of Translational Research and of New Surgical and Medical Technologies, University of Pisa, 56126 Pisa, Italy; ilaria.ambrosini@phd.unipi.it (I.A.); roberto.francischello@med.unipi.it (R.F.); francesca.caputo@med.unipi.it (F.P.C.); emanuele.neri@unipi.it (E.N.); 2 Nuclear Medicine Unit, Department of Translational Research and of New Surgical and Medical Technologies, University of Pisa, 56126 Pisa, Italy; gayane.aghakhanyan@med.unipi.it; 3 Academic Radiology, Department of Surgical, Medical, Molecular Pathology and Emergency Medicine, University of Pisa, 56126 Pisa, Italy; riccardo.lencioni@unipi.it (R.L.); dania.cioni@unipi.it (D.C.)

**Keywords:** rectal cancer, radiomics, magnetic resonance imaging, neoadjuvant therapy, machine learning, tumor regression grade, predictive modeling

## Abstract

**Background:** Response to neoadjuvant therapy in locally advanced rectal cancer (LARC) is heterogeneous, and early identification of non-responders may help optimize treatment strategies and reduce unnecessary toxicity. This study aimed to develop and internally validate a machine learning model based on radiomic features extracted from baseline magnetic resonance imaging (MRI) to predict treatment response defined according to MRI tumor regression grade (mrTRG) at restaging MRI. **Methods:** In this retrospective single-center study, 86 patients with histologically confirmed LARC who underwent baseline and restaging MRI, neoadjuvant therapy, and surgery were included. Primary tumors were manually segmented on oblique axial T2-weighted images. A total of 107 radiomic features were extracted using PyRadiomics (vrs 3.0.1), with and without N4 bias field correction. Feature selection was performed using LASSO, followed by elastic net–regularized logistic regression. Model performance was evaluated using repeated stratified 5-fold cross-validation (20 repetitions). Treatment response was defined according to MRI tumor regression grade (mrTRG) at restaging, dichotomized into responders (mrTRG ≤ 2) and non-responders (mrTRG ≥ 3). **Results:** The model achieved a mean area under the receiver operating characteristic curve (AUC-ROC) of 0.73, with an accuracy of 72.5%, sensitivity of 79.2%, and specificity of 50%. **Conclusions:** Baseline MRI-based radiomics shows potential for identifying patients at higher risk of non-response to neoadjuvant therapy in LARC. However, limited specificity and the absence of external validation restrict immediate clinical applicability. Further validation in larger multicenter cohorts and integration with clinical variables are warranted to improve model robustness and generalizability.

## 1. Introduction

Colorectal cancer (CRC) is one of the most common malignancies worldwide and a major cause of cancer-related mortality [1,2]. Rectal cancer accounts for a substantial proportion of CRC cases and shows distinct anatomical and clinical features that influence staging, treatment planning, and outcomes [3]. Although screening programs and therapeutic advances have reduced CRC incidence in older populations, recent epidemiological data indicate a growing incidence of rectal cancer in younger adults (less than 50 years old), thus emphasizing the need for improved risk stratification and personalized management [1,2,4,5].

For locally advanced rectal cancer (LARC), neoadjuvant therapies such as chemoradiotherapy (CTRT) and/or total neoadjuvant therapy (TNT) followed by total mesorectal excision (TME) represent the cornerstone of treatment, with the aim to improve local control and facilitate curative resection [6,7,8,9,10]. However, response to neoadjuvant therapy is heterogeneous, with a subset of patients showing limited or no response while still experiencing treatment-related toxicity and potential delays to definitive surgery [6,11,12,13,14]. Therefore, identifying non-responder patients before treatment could be clinically important to tailor treatment, improve prognosis stratification and reduce unnecessary exposure to ineffective therapies.

Magnetic resonance imaging (MRI) is the gold standard imaging modality for local staging of rectal cancer, providing high soft-tissue contrast and enabling assessment of key prognostic factors, including tumor extension, mesorectal fascia involvement, circumferential resection margin risk, and regional nodal involvement [15,16,17,18,19,20,21,22,23,24,25]. Moreover, MRI plays a key role also in tumor restaging after neoadjuvant therapy through the evaluation of tumor regression and residual disease [24,26,27,28,29,30]. However, conventional MRI interpretation is partly subjective and may be limited by inter-observer variability and by difficulties in distinguishing residual tumor from post-treatment changes such as fibrosis, edema, or mucinous degeneration [24,26,30,31]. In this scenario, radiomics offers a quantitative methodological approach to extract high-dimensional features from conventional radiological images, capturing patterns related to intensity, texture, shape, and intratumoral heterogeneity that are not visible by the human eye [32,33,34,35,36]. When combined with machine learning, radiomics can support predictive modeling and has shown promising results in rectal cancer for response prediction and risk stratification [32,37,38,39,40,41]. However, variability in imaging protocols, segmentation approaches, and validation strategies still limits reproducibility and generalizability, which continues to hinder clinical translation [32,33,34].

This retrospective study aimed to develop and internally validate a machine learning model based on radiomic features extracted from baseline MRI to predict response to neoadjuvant therapy, as assessed by mrTRG on restaging MRI, in LARC patients.

Response evaluation was based on MRI tumor regression grade (mrTRG) assessed at restaging MRI, adopted as the reference standard. It was hypothesized that baseline MRI-based radiomic analysis could facilitate early identification of patients at higher risk of non-response, thereby contributing to personalized treatment planning.

Despite promising results, radiomics-based predictive models in rectal cancer remain limited by variability in imaging protocols, lack of standardization, and insufficient external validation, which hinder their clinical translation.

## 2. Materials and Methods

### 2.1. Study Design and Population

This retrospective cohort study included patients aged ≥18 years with histologically confirmed rectal adenocarcinoma treated at a single tertiary referral center between 2017 and 2022. An initial retrospective review of examinations performed between 2017 and 2019 was performed and yielded a smaller sample size than anticipated. Therefore, as specified in the study protocol, the observation period was extended to include additional cases from subsequent years. These additional cases were not prospectively recruited, but identified retrospectively from routine clinical examinations performed after IRB approval and acquired under the same standard clinical practice as the earlier cases, thereby maintaining cohort homogeneity while increasing the sample size. The study was conducted in accordance with the Declaration of Helsinki approved by the Tuscany regional ethical committee for clinical experimentation—AREA VASTA NORD OVEST section—Protocol No. 18253, approved on 24 September 2020. Informed consent was waived due to the retrospective nature of the study.

Patients were selected according to the following criteria:

- inclusion criteria: availability of baseline staging MRI, completion of neoadjuvant therapy, availability of restaging MRI, surgical resection.
- exclusion criteria: poor image quality (e.g., motion artifacts), missing clinical data.

All patient-related data was anonymized according to privacy regulations.

The interval between baseline MRI and initiation of neoadjuvant therapy, as well as between completion of therapy and restaging MRI, followed standard institutional clinical practice, in particular, restaging MRI was performed 10–12 weeks after the completion of neoadjuvant treatment.

### 2.2. MRI Acquisition Protocol

All MRI examinations were performed using three scanners: 3T General Electric Discovery MR750 (GE Healthcare, Chicago, IL, USA) release software dv25 (GE Discovery MR750 3T), 1.5T General Electric Signa HDxt TwinSpeed 1.5T (GE Healthcare, Chicago, IL, USA) release software dv25 (GE Signa HDxt 1.5T), 1.5T Siemens Magnetom SymphonyTim (Siemens Healthineers, Erlangen, Germany).

**Figure 1:**
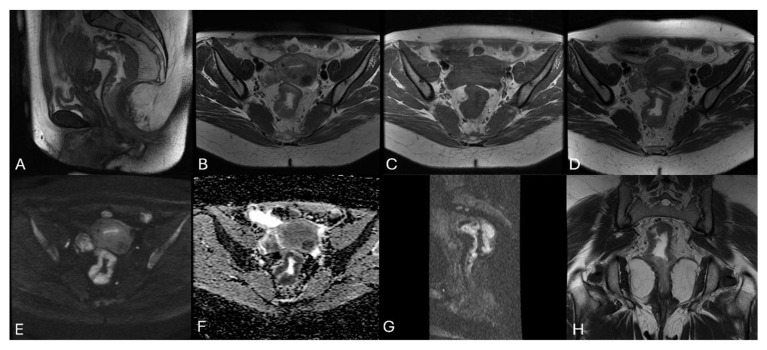
Representative images of each MRI sequence acquired for staging. (**A**) Sagittal 3D T2w; (**B**) Axial fast recovery FSE (FRFSE) T2w; (**C**) Axial T1w; (**D**) Oblique axial T2w; (**E**) Axial DWI (b-value = 1000); (**F**) Apparent diffusion coefficient (ADC) map; (**G**) Oblique coronal T2w. (**H**) Oblique coronal T2w.

Acquisition protocol included: high-resolution T2-weighted (T2w) imaging (sagittal, 3D T2w, sagittal Fast Spin Echo (FSE) T2w, axial FSE T2w, oblique axial T2w), T1-weighted (T1w) imaging (axial FSE T1w), diffusion-weighted imaging (DWI, b value > 800) (Figure 1).

Optional sequences included focus sagittal DWI (b value > 800), gradient echo (GRE) T1-weighted (T1w) in-phase (IN) and out-of-phase (OUT), oblique coronal (T2w, high resolution). Endorectal gel (60–100 mL) or intravenous antiperistaltic agents (20 mg i.v. hyoscine butyl-bromide) were administered when required.

All baseline staging MRI examinations were reviewed by a radiologist who assessed tumor location, distance of the tumor from the anal margin, TNM staging, and eventual presence of extramural vascular invasion (EMVI).

For the purposes of this study, the oblique axial T2-weighted sequence was analyzed. The sequence is acquired with an orientation perpendicular to the tumor’s major axis, thereby ensuring optimal evaluation and local staging of the primary tumor.

The acquisition parameters of this sequence for each scanner used in this study are reported in detail in Table 1.

### 2.3. MRI-Based Tumor Regression Grading System (mrTRG)

All restaging MRI examinations after neoadjuvant therapy were evaluated by a radiologist and classified according to the tumor regression grading (TRG) system, which assesses the degree of tumor regression after neoadjuvant therapy and therefore evaluates response to treatment.

TRG was primarily developed as a histopathological scoring system to evaluate tumor regression following neoadjuvant therapy, similarly to the Mandard score [31].

Subsequently, an MRI-based tumor regression grading system (mrTRG) was introduced to accurately reflect correlations between MRI after neoadjuvant therapy and pathological findings.

Similar to the Mandard system, mrTRG is a five-grade score ranging from grade 1 (absence of residual tumor or complete response (CR)) to grade 5 (no tumor regression after neoadjuvant therapy) (Table 2) [42].

In this study, mrTRG was used as an indicator of tumor regression following neoadjuvant treatment. To properly classify patients as responders or non-responders based on mrTRG, a grouping strategy was applied. Specifically, patients with mrTRG 1 or mrTRG 2 were classified as responders, whereas patients with mrTRG 3, mrTRG 4, or mrTRG 5 were classified as non-responders. This dichotomization is consistent with prior studies, where mrTRG 1–2 are generally considered responders and mrTRG ≥ 3 non-responders [45,46,47].

### 2.4. Tumor Segmentation and Pre-Processing

**Figure 2:**
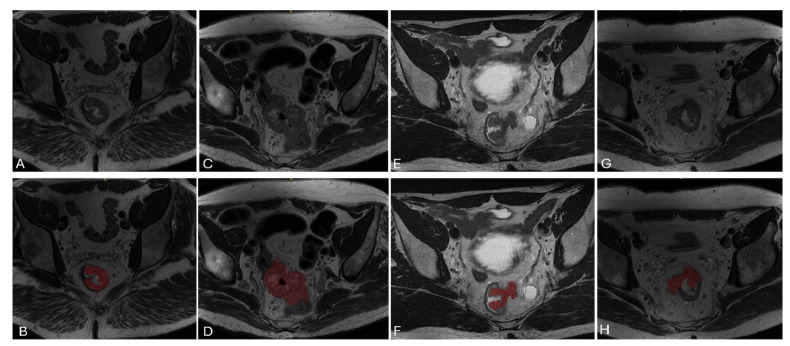
Representative examples of tumor segmentation. Native oblique axial T2-weighted images are shown in the **upper row** (**A**,**C**,**E**,**G**), with the corresponding segmentation red overlays presented in the **lower row** (**B**,**D**,**F**,**H**).

Primary tumors were manually segmented by drawing a region of interest (ROI) on baseline oblique axial MRI T2w images using an open-source software ITK-SNAP (version 3.6.0) by a junior radiologist and subsequently reviewed by a radiologist with more than ten years of experience in abdominal MRI (Figure 2). Segmentation was blinded to the study outcomes.

MRI is often affected by artifacts due to low spatial frequency intensity inhomogeneities, which may compromise the accuracy of automated image analysis algorithms, as they adversely affect the quality of automated MRI segmentation [48]. The N4 algorithm uses a parametric model, which does not rely on specific assumptions regarding the physical nature of the inhomogeneities, to estimate the impact of these artifacts on the image and to correct them [48]. To correct the images in this study, the N4BiasFieldCorrectionImageFilter function from the SimpleITK library (vrs 3.8.0) was used.

Inter- and intra-observer variability were not formally assessed in this study, which represents a limitation that should be addressed in future investigations.

### 2.5. Radiomic Feature Extraction

Radiomic features were extracted from tumor segmentation process of baseline oblique axial T2w MRI, either with and without N4 bias field correction, using open-source PyRadiomics library (vrs 3.0.1) with default settings. All images were resampled to a uniform voxel dimension (0.46875 mm, 0.46875 mm, 3.30007672 mm). The voxel side dimensions were selected as the median values of the distribution of each voxel side among all images. For texture features calculation, images were discretized into 70 gray levels. This discretization level was selected as a trade-off between preserving image intensity information and reducing noise, in line with commonly adopted radiomics practices reported in the literature.

A total of 107 features were extracted and distributed as follows: 18 first-order features; 14 shape-based features; 24 features from the Gray Level Co-occurrence Matrix (GLCM); 16 from the Gray Level Run Length Matrix (GLRLM); 16 from the Gray Level Size Zone Matrix (GLSZM); 5 from the Neighbouring Gray Tone Difference Matrix (NGTDM); and 14 from the Gray Level Dependence Matrix (GLDM).

### 2.6. Model Development and Validation

Supervised machine learning models may be prone to overfitting. This phenomenon occurs when a model is sufficiently flexible (e.g., due to a large number of parameters relative to the number of observations) to closely reproduce the training data. However, strong performance on the training set is often associated with poor generalizability, meaning reduced predictive accuracy on previously unseen data, and thereby limiting the model’s applicability in real-world settings, including clinical practice.

Although there are strategies to reduce overfitting, it is crucial to evaluate the model’s performance on a dataset not used during training. For this reason, a repeated stratified 5-fold cross-validation approach was adopted: the dataset was partitioned into five subsets (“folds”), ensuring that each fold preserved the same proportion of positive labels (mrTRG ≥ 3) and negative labels (mrTRG ≤ 2). Each fold was iteratively used as a test set while the remaining four folds constituted the training set. This partitioning procedure was repeated 20 times. At the conclusion of the process, the model’s generalization performance, defined as its ability to correctly predict unseen samples, was assessed by aggregating results across the 100 test folds to provide a robust estimate of generalization performance. No specific class balancing strategy (e.g., class weighting or resampling) was applied. This choice may have contributed to the observed imbalance between sensitivity and specificity.

The modeling pipeline consisted of three sequential steps, all performed exclusively within each training fold without access to the test data, including preprocessing, feature selection, and model tuning, to prevent data leakage. The first step involved normalization of each radiomic feature using z-score standardization (mean subtraction and division by the standard deviation). This procedure was necessary to eliminate scale-related effects that could bias the subsequent feature selection step, performed using the LASSO (Least Absolute Shrinkage and Selection Operator) method. The LASSO model was evaluated via 5-fold cross-validation and the radiomic features that maximized the mean AUC-ROC across the five folds were selected. The LASSO procedure resulted in the selection of a range of 5 to 12 radiomic features per iteration, which were subsequently used for model training.

In the final step, the LASSO-selected features were used to train a logistic regression model with elasticnet regularization. The regularization strength and L1/L2 penalty ratio were optimized using grid search, maximizing the mean AUC-ROC within a 5-fold cross-validation framework.

All analyses were performed using Python libraries (vrs 3.10), including NumPy, SciPy, pandas, and scikit-learn.

### 2.7. Statistical Analysis

Model performance was evaluated using metrics derived from the confusion matrix, including sensitivity (true positive rate, TPR), specificity (true negative rate, TNR), positive predictive value (PPV), negative predictive value (NPV), overall accuracy, and F1-score. Discriminative ability was further assessed by calculating the area under the Receiver Operating Characteristic curve (AUC-ROC). Results are reported as mean ± standard deviation across cross-validation folds.

## 3. Results

The final cohort, derived from an eligible sample of 400 individuals, included 86 patients (65% male; mean age 63 ± 12 years). The population characteristics, along with the neoadjuvant treatments administered and the mrTRG distribution, are summarized in Table 3. As only patients who underwent restaging MRI were included, most of the study population presented with locally advanced tumors. The neoadjuvant treatments included chemotherapy regimens, chemoradiotherapy, short-course radiotherapy, and induction chemotherapy.

**Figure 3:**
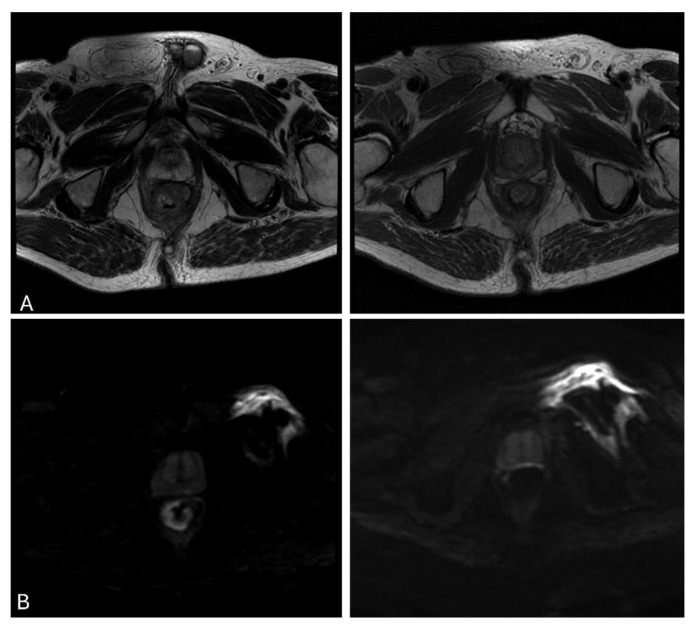
A 75-year-old male patient with low-rectal cancer, stage IIIc treated with neoadjuvant chemoradiotherapy (CTRT) and classified as a responder (mrTRG = 1). (**A**) Oblique axial T2-w image and (**B**) DWI (b-value = 1000) from the baseline examination before (**right**) and after neoadjuvant therapy (**left**).

23.26% of patients were classified as responders (mrTRG ≤ 2) based on restaging MRI (Figure 3). Consequently, 76.74% of patients were classified as non-responders on restaging MRI (mrTRG ≥ 3).

**Figure 4:**
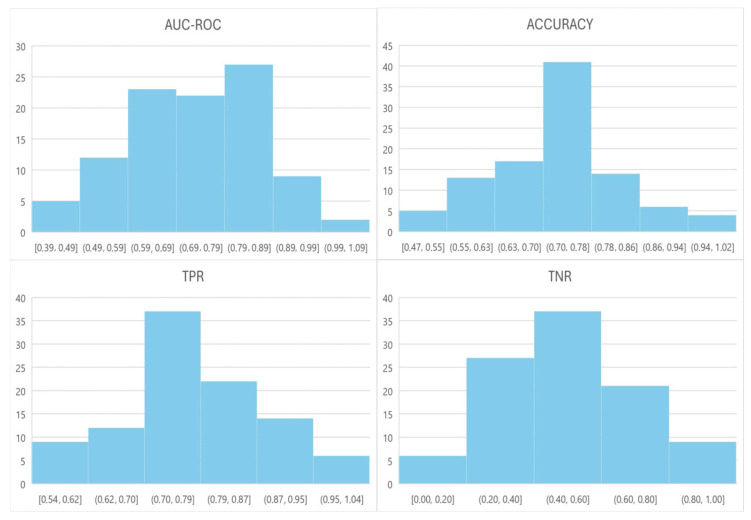
Histograms illustrating model performance across the evaluated metrics.

Model performance distributions are illustrated in Figure 4.

For each model, the Area Under the Curve (AUC-ROC), accuracy, sensitivity (true positive rate, TPR), and specificity (true negative rate, TNR) were evaluated. Subsequently, the mean values (95% CI) of all performance metrics were calculated, yielding the following results: AUC-ROC 0.73 (0.70–0.76), accuracy 0.72 (0.70–0.75), TPR 0.79 (0.77–0.81), TNR 0.50 (0.46–0.54), balanced accuracy 0.64 (0.60–0.69), PPV 0.84 (0.82–0.86), NPV 0.42 (0.36–0.49), and F1 score of 0.81 (0.79–0.84). These results indicate moderate discriminative performance.

**Figure 5:**
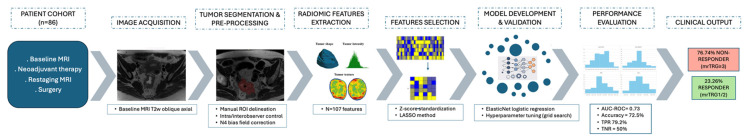
MRI-based radiomics pipeline.

Our study workflow is summarized in Figure 5.

## 4. Discussion

The present study aimed to predict non-responder patients with locally advanced rectal cancer (LARC) treated with neoadjuvant therapy using a machine learning model based on baseline MRI-derived radiomic features. The proposed model achieved a TPR of 79.2%, indicating a good sensitivity in identifying non-responders. However, the mean TNR was 50%, thus reflecting a limited ability to correctly classify responders. This imbalance suggests that while the model may be useful for detecting high-risk patients unlikely to benefit from neoadjuvant therapy, its specificity remains suboptimal. This may be significantly explained by the class imbalance of the dataset and the absence of balancing strategies during model training.

Neoadjuvant treatments, which include either chemotherapy and radiotherapy, are associated with potential toxicities that may adversely affect patient quality of life and clinical status and are not always well tolerated [39]. Accurate early identification of non-responders is therefore clinically relevant, as treatment-related adverse effects may outweigh therapeutic benefits in this subgroup.

Several prior studies have explored predictive modeling for response assessment in rectal cancer. Although sharing similar objectives, these investigations differ substantially in methodology, imaging modalities, and feature extraction strategies, highlighting the complexity of this research field. In the present study, the radiomic features were extracted exclusively from MR imaging, whereas other authors have incorporated multimodal imaging approaches, including CT and FDG PET/CT [40,46,49].

MRI represents the reference imaging modality for locoregional staging of rectal cancer, and most radiomics-based predictive models have been MRI-driven [50,51,52,53]. Nevertheless, considerable variability exists regarding the number and type of MRI sequences included. For example, a retrospective single-center study combining T2-w and T1-w images reported excellent predictive performance for pathological complete response (pCR), with an AUC of 94%, accuracy of 97%, sensitivity of 93%, and specificity of 88.9% [54].

However, integrating features from multiple imaging sources or sequences introduces methodological challenges. Beyond increased computational burden and model complexity, high-dimensional feature spaces relative to cohort size may increase the risk of overfitting [55]. To mitigate this risk, the present study employed a single MRI sequence (oblique axial T2-weighted), prioritizing model stability and interpretability. T2-weighted imaging represents the standard reference sequence for rectal cancer staging and is routinely acquired in all patients, supporting reproducibility and potential clinical translation.

Additional variability may arise from scanner-related factors. Static magnetic field strength has been suggested as a potential contributor to radiomic variability. In this study, imaging data were acquired using three MRI scanners (two 1.5T and one 3T), whereas several prior studies relied on a single scanner. No formal harmonization techniques such as ComBat were applied, and therefore scanner-related variability may have influenced radiomic feature stability. Future studies should incorporate dedicated harmonization techniques to further improve feature reproducibility across different scanners. A preliminary investigation using exclusively 3T scanners reported a higher AUC but lower sensitivity compared with the results observed in our cohort [50], suggesting that scanner homogeneity does not necessarily guarantee superior classification performance. Differences in model performance across studies may also be related to variations in segmentation strategies (e.g., whole tumor vs. border), use of single versus multiparametric MRI sequences, and choice of reference standard.

Another important methodological difference is related to the reference standard used to classify responders and non-responders. mrTRG assessment has emerged as a valuable tool within multidisciplinary oncologic management. While its non-invasive nature and repeatability offer clear advantages in repeatability and early assessment, mrTRG reproducibility may be limited with inter-reader variability, underscoring the importance of expert radiological evaluation. Furthermore, potential discrepancies between mrTRG and pathological TRG may affect response prediction accuracy, particularly when a long interval exists between MRI and surgery [44,51].

Although mrTRG enables early treatment response assessment and surgical planning, its limitations, including the frequent overestimation of residual tumor, must be acknowledged [51]. This tendency may partially explain the moderate specificity observed in our model.

Several studies have instead used histopathological TRG (pTRG) as the reference standard. For example, a retrospective study applying the AJCC pTRG classification reported comparable discriminative performance (AUC 0.77) [39] using a dichotomization strategy similar to that employed in our analysis.

Other authors have focused on predicting pCR rather than TRG categories. A retrospective study with similar inclusion criteria achieved an AUC of 0.73, although tumor segmentation was restricted to the lesion border rather than the entire tumor volume [56]. Petkovska et al. reported similar findings (AUC 0.75) when distinguishing pCR from partial pathological response [57].

Response prediction strategies also vary with respect to imaging time points. The present study focused exclusively on baseline MRI, whereas other investigations have incorporated post-treatment MRI. Li et al. reported improved performance (AUC 0.87) by combining baseline and restaging MRI features [52]. More recently, novel delta-radiomics approaches, integrating longitudinal feature changes, have shown promising results [53,58]. Differences in validation strategies further complicate comparisons across studies. While repeated stratified 5-fold cross-validation (20 repetitions) was adopted in this work to enhance robustness, other studies have used fewer repetitions or alternative validation schemes [59].

This study has several limitations that should be acknowledged. The retrospective single-center design resulted in a relatively small cohort size, which may limit statistical power. Consequently, external validation was not performed, thus limiting model generalizability. Another limitation of the present study is the heterogeneity of the administered neoadjuvant treatment regimens, including chemoradiotherapy, short-course radiotherapy, and induction chemotherapy followed by chemoradiotherapy. Treatment response may therefore have been influenced not only by intrinsic tumor biology reflected by radiomic features, but also by differences in therapeutic strategy. Furthermore, the treatment regimen was not incorporated into the predictive model as a clinical variable, which may have affected model performance and limited interpretation of the independent contribution of radiomic features.

Moreover, calibration or reproducibility analysis to evaluate feature stability (e.g., segmentation variability), as well as comparisons with clinical models, were not conducted. Future studies should assess feature robustness through test–retest and inter-observer variability analyses in order to improve reliability and generalizability. These aspects should be addressed in prospective multicenter studies. These findings should also be interpreted in the context of the broader challenges of radiomics reproducibility, as highlighted by recent methodological frameworks such as the METRICS score [45,60,61], emphasizing the need for standardized pipelines and external validation prior to clinical implementation.

## 5. Conclusions

The proposed machine learning model based on baseline MRI-derived radiomic features demonstrated good sensitivity in identifying non-responders after neoadjuvant therapy, as defined by mrTRG at restaging MRI, in patients with locally advanced rectal cancer. These findings suggest a potential role for baseline MRI radiomics in supporting early treatment stratification and more personalized therapeutic decision-making. However, limited specificity and lack of external validation currently preclude direct clinical application. Further methodological refinement and validation in larger multicenter cohorts are required before clinical implementation.

## Figures and Tables

**Table 1:** Acquisition parameters referred to the oblique axial T2-w sequence for each MRI scanner used in this study.

MRI Scanner	Echo Time (ms)	Repetition Time (ms)	Slice Thickness (mm)	FOV (mm)	Acquisition Matrix	Flip Angle
**DISCOVERY** **MR750**	61,307	1400	2	100	0/224/224/0	90
**Signa HDxt**	65	1200	2	100	0/288/256	90
**Symphony Tim**	84	4200	4	100	320/0/0/256	150

MRI: magnetic resonance imaging; FOV: field of view.

**Table 2:** mrTRG classification. This system is primarily based on comparison with baseline MRI and on the evaluation, particularly on T2w images, of the relative proportions of fibrotic and residual tumor signal intensity [43,44].

Grade	Radiologic Response	Description
mrTRG 1	Complete Response (CR)	Complete regression: no evidence of tumor signal or barely visible linear fibrotic scar (low signal intensity) in the mucosa/submucosal layer of previous tumor site
mrTRG 2	Near-complete Response (n-CR)	Good regression: predominant low signal intensity fibrotic scar with no obvious residual tumor signal
mrTRG 3	Moderate Response	Moderate regression: >50% low signal intensity fibrosis/mucin areas, but there are obvious areas of intermediate signal intensity
mrTRG 4	Mild Response	Slight regression: few areas of low signal intensity fibrosis or mucin but mostly tumor signal intensity on T2w images
mrTRG 5	No Response	Intermediate signal intensity, same appearances as the original tumor on T2w images

**Table 3:** Study population characteristics. T: tumor; N: nodal involvement; EMVI: Extramural Vascular Invasion; CT: Chemotherapy; CTRT: Chemoradiotherapy; RTSC: Short-Course Radiotherapy; CT IND > CTRT: Induction Chemotherapy followed by Chemoradiotherapy; RT: Radiotherapy; mrTRG: MRI Tumor Regression Grade.

Population Characteristics	N (%)
Age (mean ± standard deviation)	63 ± 12 years
Male	56 (65%)
Female	30 (35%)
T	T2 → 7 (8.14%)
	T3 → 8 (9.3%)
	T3a → 9 (10.47%)
	T3b → 32 (37.21%)
	T3c → 13 (15.12%)
	T3d → 3 (3.49%)
	T4 → 9 (10.47%)
	T4a → 3 (3.49%)
	T4b → 2 (2.33%)
N	N0 → 3 (3.49%)
	N1 → 27 (31.4%)
	N1b → 2 (2.33%)
	N2 → 54 (62.8%)
EMVI	absent → 55 (63.95%)
	present → 31 (36.05%)
CT	1 (1.16%)
CTRT	53 (61.63%)
RTSC	17 (19.77%)
CT IND > CTRT	14 (16.28%)
RT	1 (1.16%)
mrTRG	mrTRG1 → 1 (1.16%)
	mrTRG2 → 19 (22.09%)
	mrTRG3 → 58 (67.44%)
	mrTRG4 → 4 (4.65%)
	mrTRG5 → 4 (4.65%)

## Data Availability

The original contributions presented in this study are included in the article. Further inquiries can be directed to the corresponding authors.

## Abbreviations

The following abbreviations are used in this manuscript:

ADC	Apparent Diffusion Coefficient
AUC-ROC	Area Under the Receiver Operating Characteristic Curve
CRC	Colorectal Cancer
CR	Complete Response
CT	Chemotherapy
CTRT	Chemoradiotherapy
CT IND > CTRT	Induction Chemotherapy followed by Chemoradiotherapy
DWI	Diffusion-Weighted Imaging
EMVI	Extramural Vascular Invasion
FOV	Field of View
FSE	Fast Spin Echo
FRFSE	Fast Recovery Fast Spin Echo
GLCM	Gray Level Co-occurrence Matrix
GLDM	Gray Level Dependence Matrix
GLRLM	Gray Level Run Length Matrix
GLSZM	Gray Level Size Zone Matrix
GRE	Gradient Echo
LARC	Locally Advanced Rectal Cancer
LASSO	Least Absolute Shrinkage and Selection Operator
MRI	Magnetic Resonance Imaging
mrTRG	Magnetic Resonance Imaging Tumor Regression Grade
NGTDM	Neighbouring Gray Tone Difference Matrix
NPV	Negative Predictive Value
pCR	Pathological Complete Response
PPV	Positive Predictive Value
ROI	Region of Interest
RT	Radiotherapy
RTSC	Short-Course Radiotherapy
TME	Total Mesorectal Excision
TNT	Total Neoadjuvant Therapy
TNM	Tumor–Node–Metastasis Staging System
T1w	T1-weighted
T2w	T2-weighted
TPR	True Positive Rate
TNR	True Negative Rate
TRG	Tumor Regression Grade
